# Habitat openness and predator abundance determine predation risk of warningly colored longhorn beetles (Cerambycidae) in temperate forest

**DOI:** 10.1093/jisesa/iead027

**Published:** 2023-04-28

**Authors:** Anika Goßmann, Lucie Ambrožová, Lukas Cizek, Lukas Drag, Kostadin Georgiev, Liane Neudam, Michal Perlík, Dominik Seidel, Simon Thorn

**Affiliations:** Field Station Fabrikschleichach, Department of Animal Ecology and Tropical Biology (Zoology III), Julius Maximilians University Würzburg, Glashüttenstraße 5, 96181 Rauhenebrach, Germany; Department of Ecology, Swedish University of Agriculture, Uppsala, Sweden; Institute of Entomology, Biology Centre CAS, 370 05 Ceske Budejovice, Czech Republic; Faculty of Science, University of South Bohemia, Ceske Budejovice, Czech Republic; Institute of Entomology, Biology Centre CAS, 370 05 Ceske Budejovice, Czech Republic; Faculty of Science, University of South Bohemia, Ceske Budejovice, Czech Republic; Institute of Entomology, Biology Centre CAS, 370 05 Ceske Budejovice, Czech Republic; Field Station Fabrikschleichach, Department of Animal Ecology and Tropical Biology (Zoology III), Julius Maximilians University Würzburg, Glashüttenstraße 5, 96181 Rauhenebrach, Germany; Hessian Agency for Nature Conservation, Environment and Geology, Biodiversity center, Europastrasse 10, D-35394 Giessen, Germany; Department of Silviculture and Forest Ecology of the Temperate Zones, Georg-August-University Göttingen, Büsgenweg 1, 37077 Göttingen, Germany; Institute of Entomology, Biology Centre CAS, 370 05 Ceske Budejovice, Czech Republic; Faculty of Science, University of South Bohemia, Ceske Budejovice, Czech Republic; Department for Spatial Structures and Digitization of Forests, Georg-August-University Göttingen, Büsgenweg 1, 37077 Göttingen, Germany; Field Station Fabrikschleichach, Department of Animal Ecology and Tropical Biology (Zoology III), Julius Maximilians University Würzburg, Glashüttenstraße 5, 96181 Rauhenebrach, Germany; Institute of Entomology, Biology Centre CAS, 370 05 Ceske Budejovice, Czech Republic; Hessian Agency for Nature Conservation, Environment and Geology, Biodiversity center, Europastrasse 10, D-35394 Giessen, Germany

**Keywords:** Batesian mimicry, beetle dummies, natural disturbance, management intensification

## Abstract

Organisms have evolved different defense mechanisms, such as crypsis and mimicry, to avoid detection and recognition by predators. A prominent example is Batesian mimicry, where palatable species mimic unpalatable or toxic ones, such as *Clytini* (Coleoptera: Cerambycidae) that mimic wasps. However, scientific evidence for the effectiveness of Batesian mimicry in Cerambycids in natural habitats is scarce. We investigated predation of warningly and nonwarningly colored Cerambycids by birds in a temperate forest using beetle dummies. Dummies mimicking *Tetropium castaneum*, *Leptura aethiops*, *Clytus arietis*, and *Leptura quadrifasciata* were exposed on standing and laying deadwood and monitored predation events by birds over one season. The 20 surveyed plots differed in their structural complexity and canopy openness due to different postdisturbance logging strategies. A total of 88 predation events on warningly colored beetle dummies and 89 predation events on nonwarningly colored beetle dummies did not reveal the difference in predation risk by birds. However, predation risk increased with canopy openness, bird abundance, and exposure time, which peaked in July. This suggests that environmental factors have a higher importance in determining predation risk of warningly and nonwarningly colored Cerambycidae than the actual coloration of the beetles. Our study showed that canopy openness might be important in determining the predation risk of beetles by birds regardless of beetles’ warning coloration. Different forest management strategies that often modify canopy openness may thus alter predator–prey interactions.

## Graphical abstract

**Figure:**
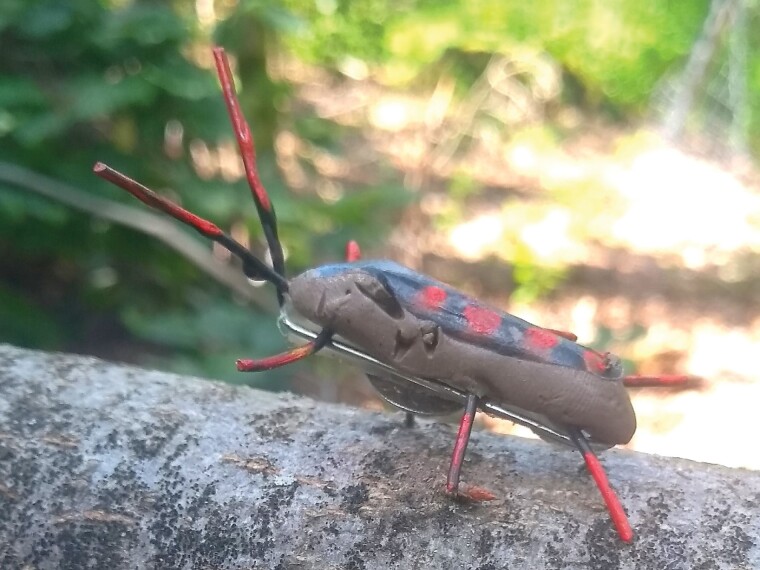
1. We investigated predation of different warningly and nonwarningly colored Cerambycids by birds in a temperate forest using beetle dummies. 2. We found no difference in predation risk by birds between warningly colored and nonwarningly colored beetle dummies. However, predation risk increased with canopy openness, bird abundance, and exposure time. 3. Canopy openness plays a crucial role in determining the predation risk of beetles by birds. Different forest management strategies modify canopy openness that can alter predator–prey interactions.

## Introduction

Avoiding predator attacks has resulted in a wide range of various defensive strategies (Emlen 1966, Charnov et al. 1976, Ruxton et al. 2019). Among them, camouflage is a widespread strategy and can be divided into background matching, where coloration of a potential prey species resembles the background, and disruptive coloration, where patterns break up the appearance and body outline of an animal (Ruxton et al. 2019). However, defensive coloration combined with harmfulness can also indicate preys’ unpalatability to a potential predator, that is, aposematism, based on innate or learned color avoidance of predators (Ruxton et al. 2019). A famous example is the black and yellow coloration of bees and wasps combined with their venomous sting. Nevertheless, aposematic animals can experience high rates of predation due to their conspicuousness, especially at low population densities and in the presence of naive predators (Lindström et al. 2001a). Thus, detectability, that is, probability of prey being found, may increase with increasing conspicuous coloration, leading to a higher risk of being attacked by a naive consumer (Endler et al. 1988, Lindström et al. 1999). Furthermore, acceptability, that is, probability of prey being attacked after detection, may depend on the predator community and prey species character (Hunter 2000, Lindström et al. 2001b).

Besides camouflage and aposematism, two major forms of defense mechanisms have evolved taking advantage of aposematic coloration—Müllerian and Batesian mimicry. In Müllerian mimicry, 2 or more unpalatable prey species mimic each other’s honest warning coloration to their mutual benefit (Ruxton et al. 2019). This was found in, for instance, bees (Hymenoptera) forming a so-called mimicry ring (Dressler 1979). In Batesian mimicry, members of a palatable species aim on decreasing their predation risk by mimicking an unpalatable species (Ruxton et al. 2019). Bates (1862) noted that if predators attack an unpalatable species first, they will avoid this species and its palatable mimics in subsequent encounters. Batesian mimicry depends on the interaction between a palatable species that mimics the warning signals of an unpalatable species and the avoidance learning of a potential predator towards warning signals. A prominent example of Batesian mimicry includes the tribe *Clytini* (Coleoptera: Cerambycidae) that mimic wasps by black and yellow coloration (Linsley 1959a).

Besides prey coloration, habitat structure can affect predation risk and thus influence habitat choice of prey. Structural habitat complexity can act as a physical impedance to the search efficiency of predators and thus lower predation risk of prey. This phenomenon is described in the total-foliage hypothesis, which suggests a decreasing predator efficiency with increasing vegetation density or structural heterogeneity due to inhibition of visual, olfactory, and auditory cues emitted by prey (Chalfound and Martin 2009). Furthermore, habitat structures provide refuges for prey and hinder the movement of predators, what results in a decrease of prey mortality (Klecka and Boukal 2014). Contrastingly, habitat structures can provide cover for ambush predators and thus increase the predation of prey (Cresswell et al. 2010).

However, most existing studies on predation in differently structured forests were conducted with nonwarningly colored prey (Maas et al. 2015, Roels et al. 2018) or warningly colored caterpillars (Tullberg et al. 2005, Remmel and Tammaru 2009). Hence, in situ quantifications of the relative importance of the effects of habitat structure and Batesian mimicry in beetles are lacking.

We investigated bird predation on warningly and nonwarningly colored Cerambycidae dummies exposed at different levels of habitat complexity over an entire growing season. We hypothesized that (i) predation risk is higher for nonwarningly colored beetles compared with warningly colored beetles and (ii) predation risk of warningly and nonwarningly colored beetles increases with decreasing habitat complexity.

## Materials and Methods

### Study Area and Experimental Design

**Fig. 1.**
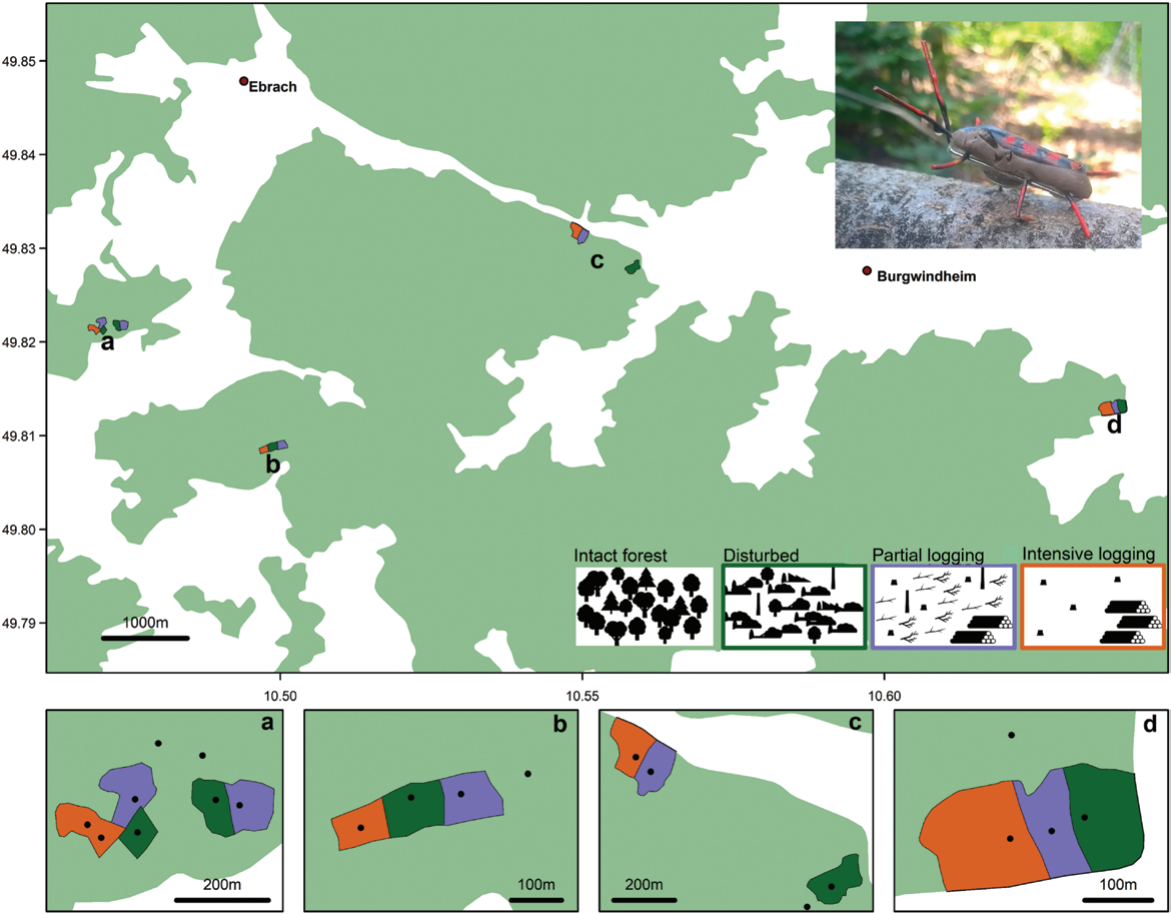
Distribution of 5 study plots in Steigerwald forest (Germany) across naturally disturbed forests with various postdisturbance management (intact forest, disturbed, partially logged, and intensively logged). Top right shows hacking beak marks on a *Leptura quadrifasciata* dummy.

Our study was conducted in the Steigerwald forest, located in northern Bavaria, Germany (N 49° 50ʹ; E 10° 29ʹ). This area covers around 16.500 ha of mixed forests with a mean annual temperature of 7–8 °C and a mean annual precipitation of 750–850 mm (Enders 1996). Here, forest stands are dominated by 44% European beech (*Fagus sylvatica* L.), 20% Sessile oak (*Quercus petraea* (Matt.) Liebl.), and 14% Scots pine (*Pinus sylvestris* L.). Forest management in the study area is based on selective cutting and a closed-canopy cover forestry (Roth et al. 2018). To cover a gradient in forest structures and canopy openness, we selected naturally disturbed stands with various postdisturbance management (Fig. 1).

In September 2018, the storm “Fabienne” passed through the study area and damaged around 40.000 m^3^ of deciduous wood, that is, trees, which died through disturbance or the following intervention. Every damaged area included plots that were left unlogged, salvage logged partially (only the main trunks removed), or intensively (all wood of diameter > 7 cm) (Fig. 1). Together with intact stands, these treatments resulted in a gradient of canopy openness and structural complexity.

In total, we established 20 plots covering the 4 treatments, that is, intact forest, disturbed and unlogged forest, partially logged forest, and intensively logged forest (Fig. 1). The experimental plots were distributed among 4 blocks. Three blocks encompassed one replicate of each treatment, while one block encompassed 2 replicates (Fig. 1). The plot size was around 1.5 ha on average.

### Beetle Dummies

**Fig. 2.**
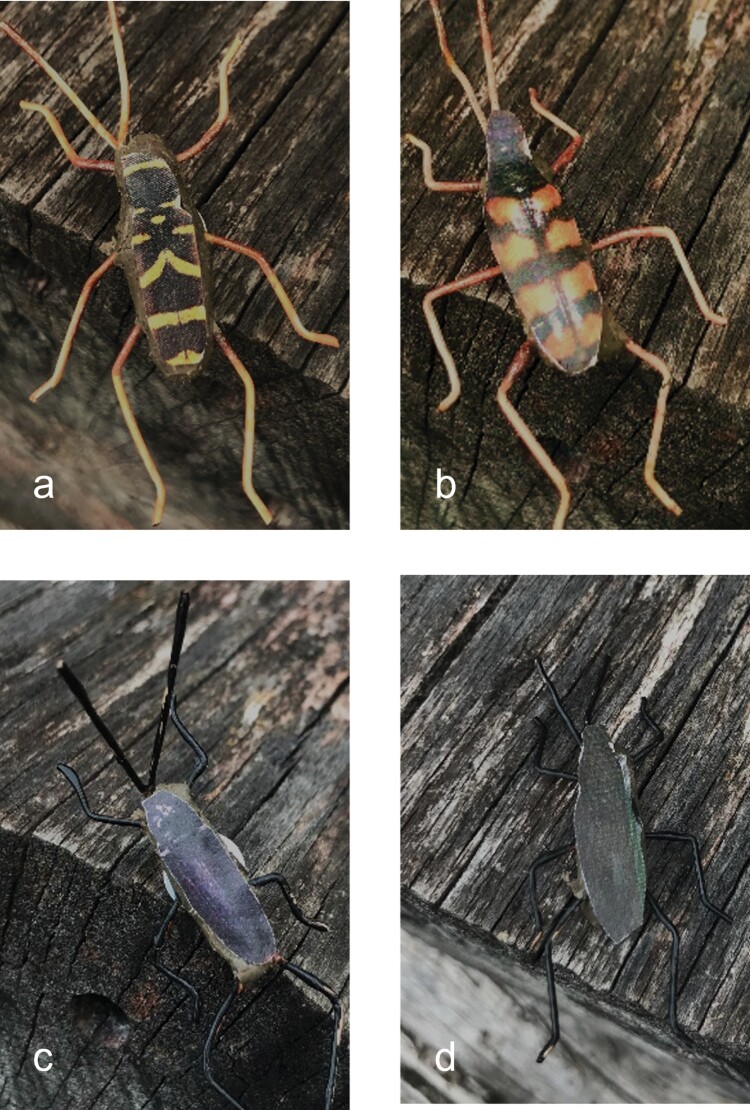
Warningly and nonwarningly colored beetle dummies: a) *Clytus arietis*, b) *Leptura quadrifasciata*, c) *Tetropium castaneum*, d) *Leptura aethiops*.

Dummies were made of paperclips with wires forming antennae and legs that were painted with nail polish. On top, we used a thin layer of plasticine and printed cover picture of the respective study species (Fig. 2).

The dummies represented 4 beetle species. Two warningly colored, that is, *Clytus arietis* (Linnaeus, 1758) and *Leptura quadrifasciata* (Linnaeus, 1758) (Fig. 2a and b), and 2 nonwarningly colored, that is, *Tetropium castaneum* (Linnaeus, 1758) and *Leptura aethiops* (Poda, 1761) (Fig. 2c and d).

On each study plot, we placed 4 dummies representing the 4 beetle species on trunks and snags with a distance of >2 m between each other. Independent of the different treatment types, we placed the dummies on similar structures, for instance, tree stumps or branches with a maximum 1 m above ground. Thus, a total of 80 dummies was exposed during the survey campaign. The dummies were exposed from mid-May until the beginning of September 2020.

Each dummy was controlled for beak marks of predation by birds. Beak marks in the plasticine had a triangular form with 1–2 cm in length and around 0.5 cm depth into the plasticine. Marks that could not be identified as predation by birds were not considered as predation. The controls took place in time intervals between 1 and 14 days (Supplementary Fig. 1). During each control, beetle dummies were relocated (min. 2 m distance from the previous location) to a different position on each study plot. In case of a previous bird predation, dummies were repaired before their relocation. This procedure resulted in a total of 1,360 possible predation events over the entire season.

### Environmental Variables

We conducted point-stop count surveys with a radius of 50-m around the center of a plot to record breeding bird communities (Bibby et al. 1992). We restricted our data analysis to insectivorous birds only, according to the feeding guild provided by Glutz von Blotzheim and Bauer (1966). Hymenoptera, representing the templates for warning colors, were sampled with 2 flight-interception traps placed at the center of each plot. Flight-interception traps consisted of a crossed pair of transparent plastic shields with size 40 cm × 60 cm. Traps were exposed from April to September and emptied monthly. We pooled trapped specimens of the genera *Vespula*, *Polistes*, *Dolichovespula*, *Bombus*, and *Apis* to yield abundances of warningly colored Hymenoptera.

To measure stand structural complexity and canopy openness, a 3D terrestrial laser-scan was conducted in 2020 on every plot in single-scan mode using a Faro Focus M70 device (Faro Technologies Inc., Lake Marry, USA). The scanner was mounted on a tripod at breast height (1.3 m) and operated with an angular scan resolution of 0.035° and a maximum scan range of 70 m. From each scan we calculated the canopy openness following the approach introduced by Zheng et al. (2013) but using a 60° opening angle and the structural complexity using the laser-scanning-based stand structural complexity index introduced by Ehbrecht (2017, 2021) using Mathematica software (Wolfram Research Inc., USA).

### Data Analysis

All data analyses were carried out in R version 4.0.2 (R Core Team 2021). Prior to statistical analysis, we corrected the structural complexity by canopy openness to account for their colinearity, that is, plots with high canopy openness had lowest structural complexity. Therefore, we fitted a linear model with structural complexity as a response variable and canopy openness as a predictor. The residual complexity was afterwards used for statistical modeling. We modeled the predation risk via a generalized additive mixed model for binomial data, provided by the package “mgcv” (Wood 2017). We selected predated/not predated as a binary response variable and exposure time, abundance of warningly colored Hymenoptera, bird abundance, warning coloration (yes/no), canopy openness, and residual structural complexity as predictors. We did not estimate species-specific slopes due to nonsufficient number of observations. Furthermore, we added the Julian date as a spline smooth term by means of the function *s*. To account for our nested study design, we added the experimental block, plot-identity, bird survey month, and treatment as random effects, too. Multi-colinearity below a threshold of 0.5 among predictor variables was ensured by variance inflation factors by means of the function *vifstep*, “usdm” package (Naimi et al. 2014).

## Results

We recorded a total of 88 predation events on warningly colored beetle dummies and 89 events on nonwarningly colored dummies, resulting in a total of 13.01% predated beetle dummies that were attacked by birds. This corresponds to a mean of 1.9% (SD: ±4.4%) of bird attacks per day. Most of the warningly and nonwarningly colored beetle dummies were predated within an exposure time of 6 days (Supplementary Figs. 2–4, Supplementary Table 1).

**Fig. 3.**
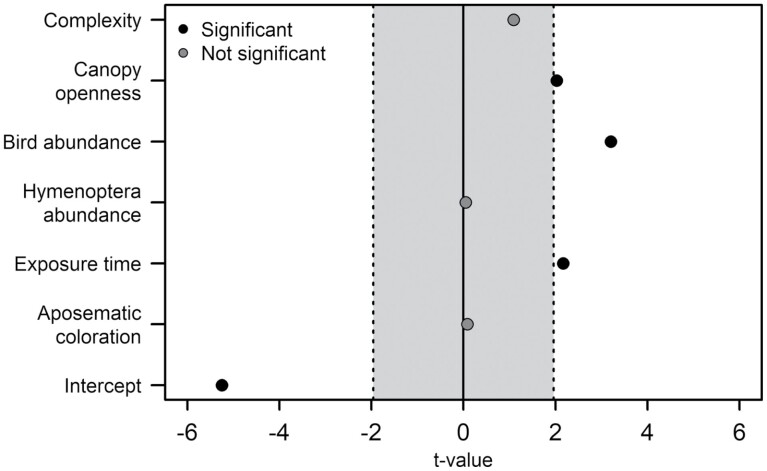
Effect of warning coloration, complexity, canopy openness, exposure time, bird abundance, and Hymenoptera abundance on predation risk based on generalized additive models. Vertical lines indicate range of nonsignificant values (−1.96 < *t*-value < 1.96).

The warning coloration had no significant effect on predation risk (*P*-value = 0.93, Fig. 3, Supplementary Table 2). Predation risk increased with increasing canopy openness (*P*-value = 0.042, Fig. 3, Supplementary Table 3, Supplementary Fig. 5) and exposure time (*P*-value = 0.03, Fig. 3, Supplementary Table 3) of the dummies. Furthermore, predation risk increased with increasing bird abundance (*P*-value = 0.001, Fig. 3, Supplementary Tables 2 and 3, Supplementary Fig. 6). Structural habitat complexity and warningly colored Hymenoptera abundance had no effect on predation risk (Fig. 3, Supplementary Tables 2 and 3).

**Fig. 4.**
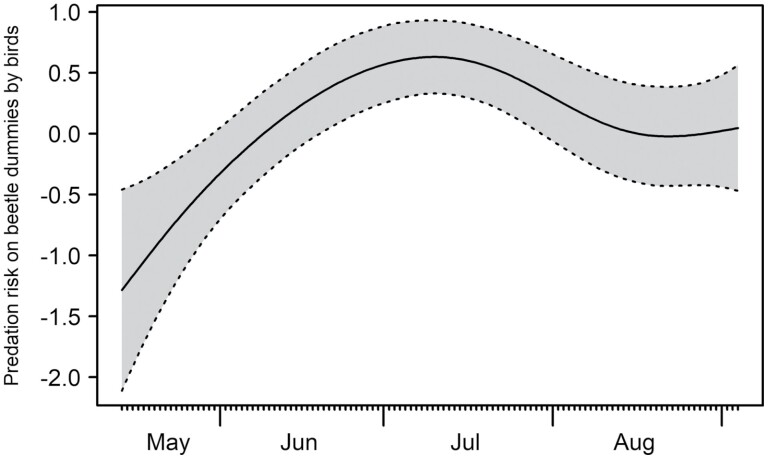
Partial effect of predation risk on beetle dummies by birds across season, modeled by generalized linear mixed models. The gray-shaded area indicates the confidence interval.

Predation risk (partial effect from generalized additive mixed models) increased from mid-May over the course of the season and peaked in July (*P*-value = 0.0008). We recorded a slight decrease in predation risk in August (Fig. 4).

## Discussion

Predation risk increased with increasing exposure time and bird abundance. Similarly, habitat structure affected predation risk by birds on beetle dummies positively, resulting in higher predation with increasing canopy openness. By contrast, warning coloration of beetle dummies had no effect on predation risk with a similar predation on warningly and nonwarningly colored beetle dummies.

During the study period, 13.01% of beetle dummies were attacked by birds with a daily mean predation rate of 1.9% of beetle dummies. For comparison, Remmel et al. (2011) found a daily mean predation rate of 3.1% on insect larvae by birds. According to our expectations, bird abundance was a significant predictor of predation risk. This is in line with Roels et al. (2018), who found a strong positive correlation between bird abundance and bird attacks on dummy caterpillars in tropical forests. Similarly, Van Bael et al. (2008) showed a strong positive correlation between bird abundance and predation on arthropods in a tropical agroforestry system.

### Predation Risk Varies Across Season

Over the surveyed months, predation risk increased significantly and peaked in July (Fig. 4). Before young birds start foraging by themselves, non-naivety and feeding habit of adult birds for their nestlings could explain the lower predation risk on beetle dummies in mid-May. For instance, young tits are mainly fed with caterpillars and spiders (Exnerová et al. 2006), and also nestlings of different species of passerine birds were mostly fed with Lepidoptera larvae (Sanz 1998) due to their high-quality nutritional profile (Arnold et al. 2010). Hernández-Agüero et al. (2020) documented a similar seasonal pattern with highest attack rates of birds on caterpillars during summertime (June–September). During these months, juvenile birds become independent and start foraging by themselves (Hernández-Agüero et al. 2020). Zvereva et al. (2021) showed that general predation risk of birds on caterpillar dummies in boreal forests increased 7-fold from early summer to midsummer, while the time of this increase coincides with the fledgling of juvenile birds. Thus, the naivety of fledglings toward prey and the overall increased number of birds due to the fledglings could explain the increase in predation risk during early summer, the predation peak in July (Fig. 4), and the similar predation risk on warningly and nonwarningly colored beetle dummies (Fig. 3).

### Importance of Habitat Structure

Predation risk of birds on beetle dummies increased with increasing canopy openness (Fig. 3). This is in line with Pough and Brower (1977), who found a higher predation intensity on butterflies by birds in an open habitat than in a wooded one. High canopy openness might increase predator–prey interaction due to the lower amount of refugia for prey (Andruskiw et al. 2008). Thus, birds might prefer open vegetation for foraging, where prey is better visible (Blake and Hoppes 1986). In contrast, Maas et al. (2015) found no difference in predation risk of birds on caterpillar dummies under different local shade-tree management in Indonesian cacao plantations. Another study from the Neotropics showed a higher predation rate of birds on caterpillar dummies in shaded coffee agroecosystems (Perfecto et al. 2004). This mixed study indicates that the effect of canopy openness on predation rate is largely context dependent. Hence, the effect of habitat structure on predation risk might be targeted by future research.

### Lacking Effect of Warning Coloration

We did not detect an effect of warning coloration of beetle dummies on the risk of predation by birds, which can be explained by several approaches.

The effects of detectability and acceptability could have balanced each other, leading to no difference in predation risk on warningly and nonwarningly colored beetle dummies. Remmel and Tammaru (2009) showed that mortality risk of insect prey is determined by the probability of being detected by a predator rather than by coloration. However, they concluded that the interaction of detectability and acceptability is highly context dependent (Remmel and Tammaru 2009).

Furthermore, in our study, mimicry could be less effective since the warning coloration was not accompanied by other, for example, behavioral components, leading to a higher acceptability by predators. Some warningly colored beetles mimic wasp coloration and movement patterns (Linsley 1959b). For instance, the *Clytini* (Coleoptera: Cerambycidae) mimic wasps by their warning coloration and in their behavior, that is, running over logs and branches in the sunlight (Linsley 1959b).

When it comes to acceptability by predators, various bird species predate warningly colored insects irrespective of their coloration and defensive secretion (Brower 1988). Some predators are resistant or highly tolerable toward noxious chemicals. Thus, there is consequently no need of learning the signal of warning coloration (Brower 1988). For instance, blackbirds *Turdus merula* (Linnaeus, 1758) showed no signs of nausea after consumption of experimentally exposed firebugs. In our study, more tolerable bird species could have predated warningly colored beetle dummies.

In contrast, various bird species cannot learn the signal of warning coloration at all. Especially granivorous birds, whose diets include mostly seeds, attack insects regardless of their coloration (Exnerová et al. 2003). This could be explained by their naivety and reduced ability to recognize and learn warning signals.

We classified beetle dummies according to their human perception, that is, visible wavelength. Even though bird species are often able to detect wavelengths beyond human perception (Cutthill et al. 2000), previous studies relying on human vision images received acceptable results (Smith 1980, Schuler and Hesse 1985, Howe et al. 2009).

In conclusion, we found a lacking effect of warning coloration, which might be driven by a balancing effect of detectability and acceptability, missing behavioral components of beetle dummies and bird species that cannot learn the signals of warning coloration or are tolerable toward noxious prey. However, we showed that predation risk of beetles by birds increases with bird abundance, advance of vegetation season, and canopy openness. Different forest management strategies modify canopy openness. Thus, we concluded that these management strategies can alter predator–prey interactions.

## Supplementary Material

iead027_suppl_Supplementary_Information

## Data Availability

The data that support the findings of this study are available from the corresponding author upon reasonable request.
