# Supplementary material for: Habitat openness and predator abundance determine predation risk of warningly colored longhorn beetles (Cerambycidae) in temperate forest
Source: J Insect Sci. 2023 Apr 28;23(2):16. doi: 10.1093/jisesa/iead027 (PMC10146197; doi:10.1093/jisesa/iead027)
Supplement: iead027_suppl_Supplementary_Information [file iead027_suppl_supplementary_information.docx]

**Habitat openness and predator abundance determine predation risk of warningly coloured long-horn beetles (Cerambycidae) in temperate forest**

Anika Goßmann, Field Station Fabrikschleichach, Department of Animal Ecology and Tropical Biology (Zoology III), Julius Maximilians University Würzburg, Glashüttenstraße 5, 96181 Rauhenebrach, Germany. Swedish University of agriculture, Dept. of Ecology, Uppsala, Sweden. [Anika.gossmann@slu.se](mailto:Anika.gossmann@slu.se), +46724459458.

Lucie Ambrožová, Institute of Entomology, Biology Centre CAS, 370 05, Ceske Budejovice, Czech Republic & Faculty of Science, University of South Bohemia, Ceske Budejovice, Czech Republic.

Lukas Cizek, Institute of Entomology, Biology Centre CAS, 370 05, Ceske Budejovice, Czech Republic & Faculty of Science, University of South Bohemia, Ceske Budejovice, Czech Republic.

Lukas Drag, Institute of Entomology, Biology Centre CAS, 370 05, Ceske Budejovice, Czech Republic & Faculty of Science, University of South Bohemia, Ceske Budejovice, Czech Republic.

Kostadin Georgiev, Hessian Agency for Nature Conservation, Environment and Geology, State Institute for the Protection of birds, Europastrasse 10, D-35394 Giessen & Field Station Fabrikschleichach, Department of Animal Ecology and Tropical Biology (Zoology III), Julius Maximilians University Würzburg, Glashüttenstraße 5, 96181 Rauhenebrach, Germany.

Liane Neudam, Georg-August-University Göttingen, Department of Silviculture and Forest Ecology of the Temperate Zones, Büsgenweg 1, 37077 Göttingen, Germany.

Michal Perlík, Institute of Entomology, Biology Centre CAS, 370 05, Ceske Budejovice, Czech Republic & Faculty of Science, University of South Bohemia, Ceske Budejovice, Czech Republic.

Dominik Seidel, Georg August University Göttingen, Department for Spatial Structures and Digitization of Forests, Büsgenweg 1, 37077 Göttingen, Germany.

Simon Thorn, Hessian Agency for Nature Conservation, Environment and Geology, State Institute for the Protection of birds, Europastrasse 10, D-35394 Giessen & Field Station Fabrikschleichach, Department of Animal Ecology and Tropical Biology (Zoology III), Julius Maximilians University Würzburg, Glashüttenstraße 5, 96181 Rauhenebrach, Germany & Institute of Entomology, Biology Centre CAS, 370 05, Ceske Budejovice, Czech Republic.

**Supplementary Information**


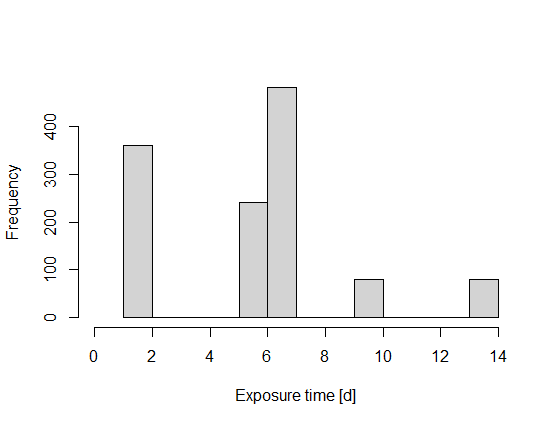


Supplementary Figure 1: Frequency of exposure time.


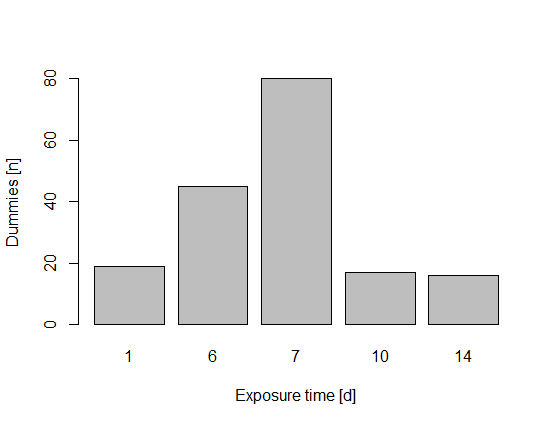


Supplementary Figure 2: Number of predated dummies in different exposure times.


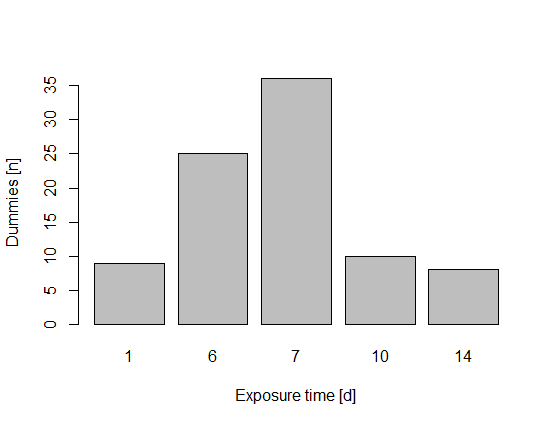


Supplementary Figure 3: Number of predated aposematic coloured dummies in different exposure times.


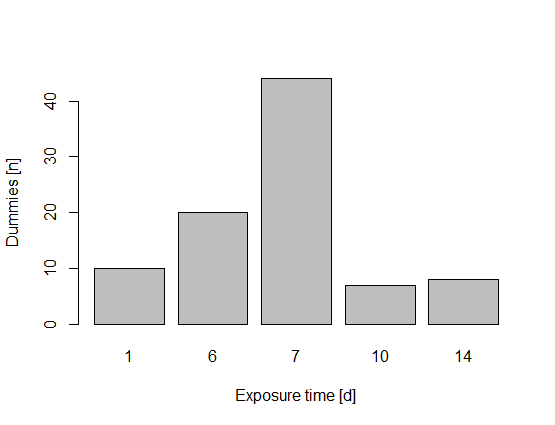


Supplementary Figure 4: Number of predated non-aposematic coloured dummies in different exposure times.

Supplementary Table 1: Predation of warningly and non-warningly coloured dummies on different treatments.

| **Dummy type** | **Intact forest** | **Disturbed forest** | **Partially logged** | **Intensively logged** |
| --- | --- | --- | --- | --- |
| **Warningly coloured** | 20.45% | 35.22 % | 27.27 % | 17.05 % |
| **Non-warningly coloured** | 20.22 % | 28.09 % | 29.21 % | 22.47 % |

Supplementary Table 2: Minimum and maximum, mean and standard deviation of bird and hymenoptera abundance on the study sites.

| **Explanatory variable** | **Study site** | **Min. value** | **Max. value** | **Mean** | **SD** |
| --- | --- | --- | --- | --- | --- |
| **Bird abundance** | a | 12 | 28 | 20.63 | 6.29 |
|  | b | 7 | 28 | 20.75 | 8.24 |
|  | c | 12 | 38 | 23.25 | 10.45 |
|  | d | 11 | 41 | 27.5 | 13.69 |
| **Hymenoptera abundance** | a | 0 | 67 | 13.55 | 17.49 |
|  | b | 0 | 85 | 11.5 | 26.56 |
|  | c | 0 | 10 | 3.9 | 3.09 |
|  | d | 1 | 179 | 43.12 | 43.68 |

Supplementary Table 3: Model outcome with bird predation as response variable.

|  | **Estimate** | **SE** | ***t*-value** | ***p*-value** |
| --- | --- | --- | --- | --- |
| **Warning colour** | 1.42$e^{-2}$ | 0.169 | 0.084 | 0.933 |
| **Exposure time** | $7.25e^{-2}$ | 0.033 | 2.170 | **0.030 *** |
| **Warningly coloured Hymenoptera abundance** | $6.67e^{-3}$ | 0.141 | 0.047 | 0.962 |
| **Bird abundance** | 1.074 | 0.336 | 3.202 | **0.001 **** |
| **Structural complexity** | 0.0119 | 0.006 | 2.027 | **0.043 *** |
| **Canopy openness** | 0.179 | 0.164 | 1.090 | 0.276 |

**
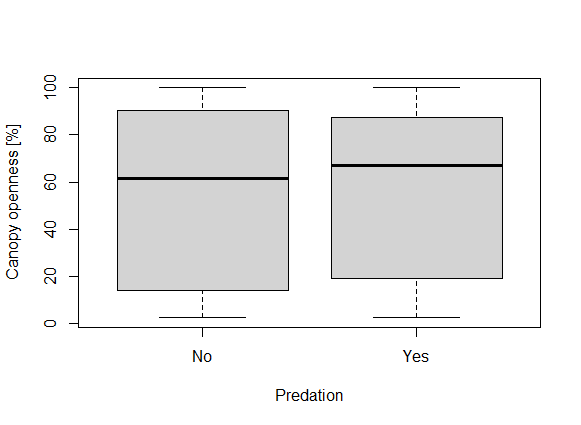
**

Supplementary Figure 5: No predation and predation of beetle dummies in relation to canopy openness.

**
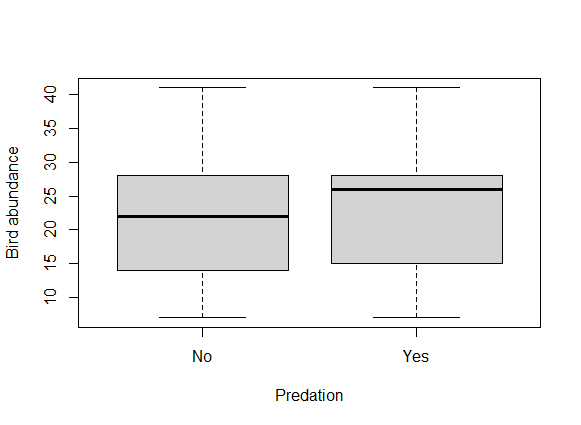
**

Supplementary Figure 6: No predation and predation of beetle dummies in relation to bird abundance, i.e. number of individuals.
